# Supplementary material for: Electron transport chain capacity expands yellow fever vaccine immunogenicity
Source: EMBO Mol Med. 2024 May 14;16(6):1310–23. doi: 10.1038/s44321-024-00065-7 (PMC11178804; doi:10.1038/s44321-024-00065-7)

Mok et al.: Electron transport chain capacity expands yellow fever vaccine immunogenicity

Appendix

Appendix Fig S1 – Overview of YFMET clinical trial design (page 2)

Appendix Fig S2 – Lower rate of symptomatic outcome observed in this study was associated with reduced YF17D antigenic load per vaccine dose compared to those used by us previously (page 3)

Appendix Fig S3 – Elevated anti-viral responses at pre vaccination (D0) baseline in subjects with undetectable viremia compared to detectable subjects (page 4)

Appendix Fig S4 – IFNγ T cell response against YF17D were not different between placebo and metformin subgroups (page 5)

Appendix Fig S5 – Cytokine expression following YF17D vaccination was not significantly different between placebo and metformin subgroups (page 6)

Appendix Fig S6 – Metformin treatment did not alter cell frequency of innate and adaptive immune cells (page 7)

Appendix Fig S7 – GSEA pre-ranking of differentially enriched pathways in metformin vs placebo subgroups (page 8)

Appendix Fig S8 – Expression levels of endoplasmic reticulum (ER) and ribosomal subunit genes at D3 post-vaccination were correlated with eventual YF17D neutralizing antibody response (page 9)

Appendix Fig S9 – Expression levels of mitochondrial respiratory complex genes at D3 post-vaccination were correlated with eventual YF neutralizing antibody response (page 10)

Appendix Fig S10 – Metformin treatment did not alter plasma levels of glucose and glycolytic intermediates upstream of pyruvate (page 11)

Appendix Table S1 – Demographics of all recruited subjects (page 12)

Appendix Table S2 – List of cytokine and chemokines measured (page 13)

Appendix Table S3 – List of immunoglobulin genes analyzed (page 14)

Appendix Supplemental Information - CONSORT Flow diagram (page 15)

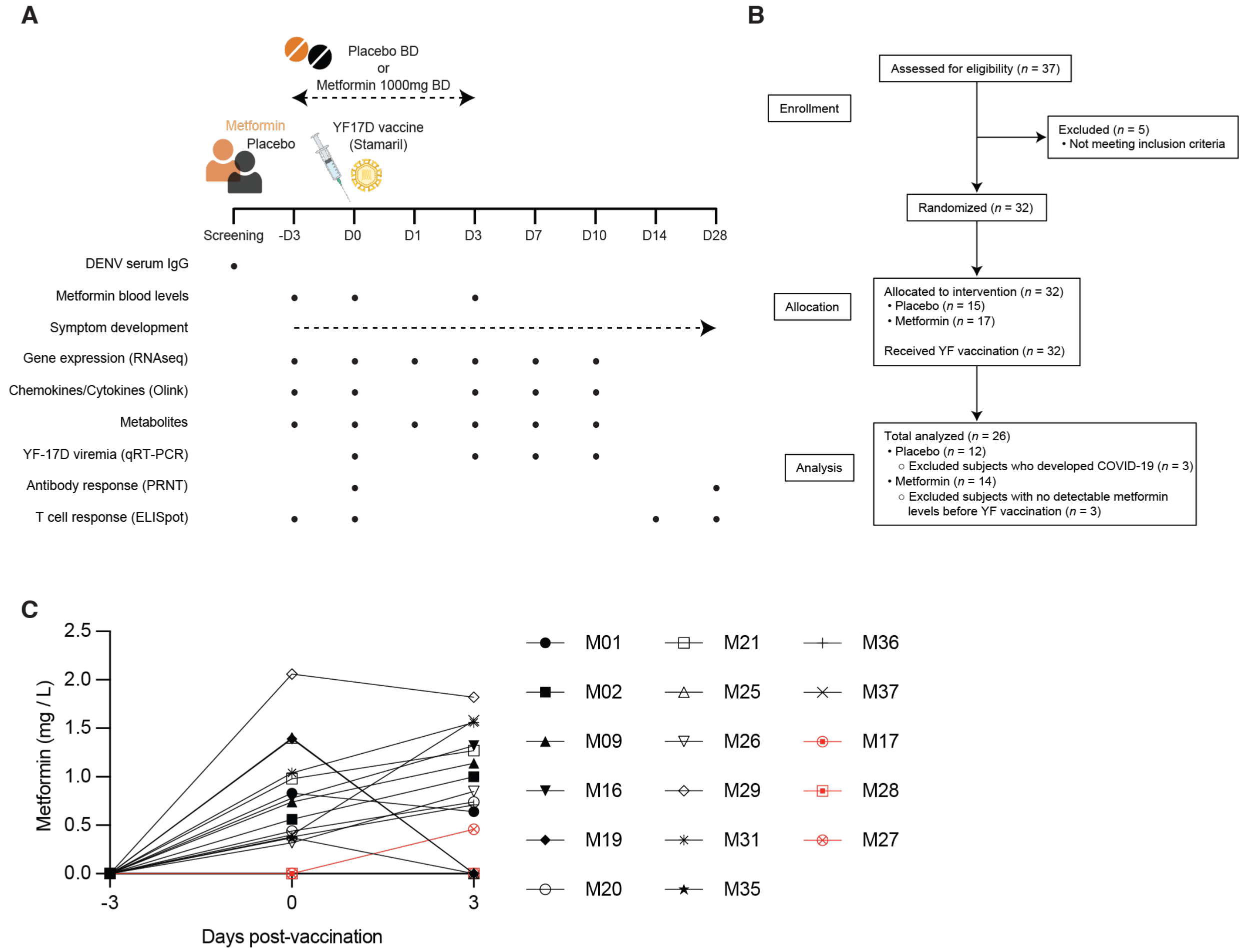

Appendix Figure S1 – Overview of YFMET clinical trial design.

A Diagrammatic representation of the study. Healthy adults were pre-screened for anti-dengue IgG antibodies before recruitment. Recruited volunteers were randomized 1:1 to receive either placebo (vitamin D/calcium tablets) or 1000mg metformin twice daily from 3-days before to 3-days after live-attenuated YF17D vaccination (Stamaril). Subjects were monitored during the trial period for symptom development, and bloods were collected at the indicated timepoints for analysis of gene expression, cytokines, metabolites, viremia, and adaptive immune responses.

B CONSORT diagram illustrating the flow of participants through each stage of the trial.

C Plasma concentrations of metformin measured at -D3, D0, and D3 post-vaccination in the metformin group Subjects highlighted in red have no measurable levels of metformin at D0 before YF17D vaccination.

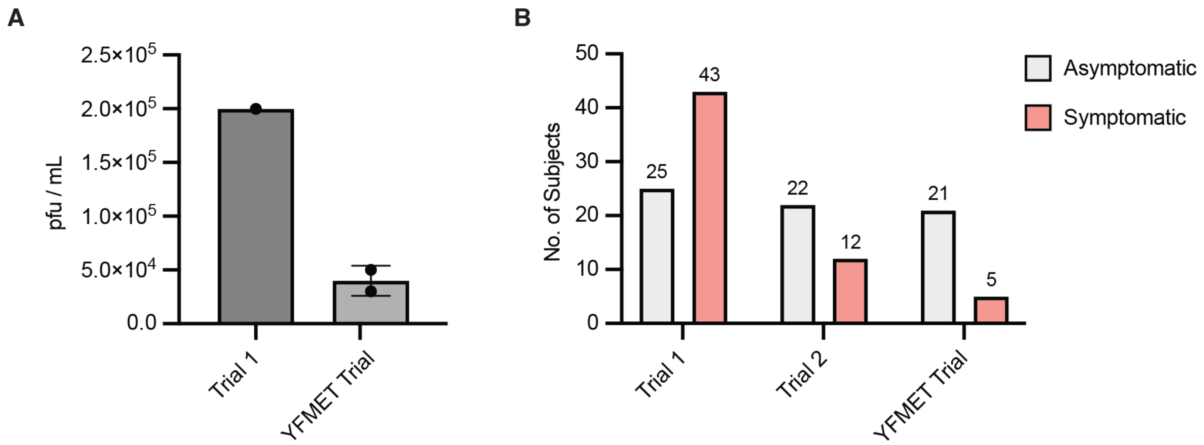

Appendix Figure S2 – Lower rate of symptomatic outcome observed in this study was associated with reduced YF17D antigenic load per vaccine dose compared to those used by us previously.

A Plaque titers of YF17D vaccines used in a previous study (Trial 1) and the current (YFMET Trial) study.

B Number of subjects with or without symptoms following YF17D vaccination in two previously conducted studies in our lab [Trial 1: Chan et al, JCI Insight (2017); Trial 2: Chan et al, Nat Med (2019)] and the current YFMET trial.

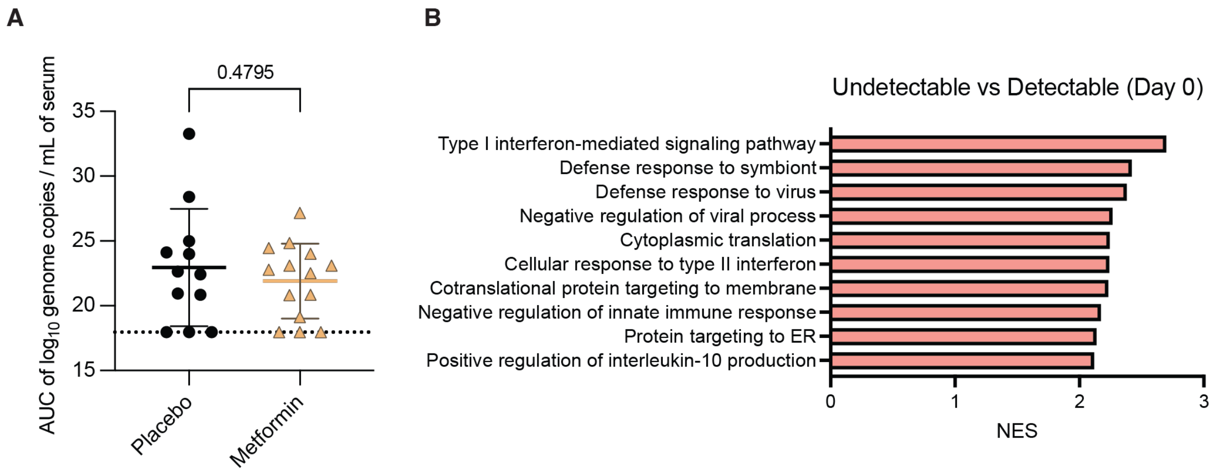

Appendix Figure S3 – Elevated anti-viral responses at pre vaccination (D0) baseline in subjects with undetectable viremia compared to detectable subjects.

A Area under the curve (AUC) of YF17D viremia in placebo treated and metformin treated subjects. The dotted line represents the lower limit of detection of the assay.

B Top 10 positively and significantly enriched Gene Ontology Biology Process (GOBP) pathways identified by pre-ranked GSEA in subjects with undetectable compared to detectable viremia at D0 before YF17D vaccination.

Data information: In (A), data are represented as mean ± SD, and statistical analyses were performed with unpaired Student’s t-test.

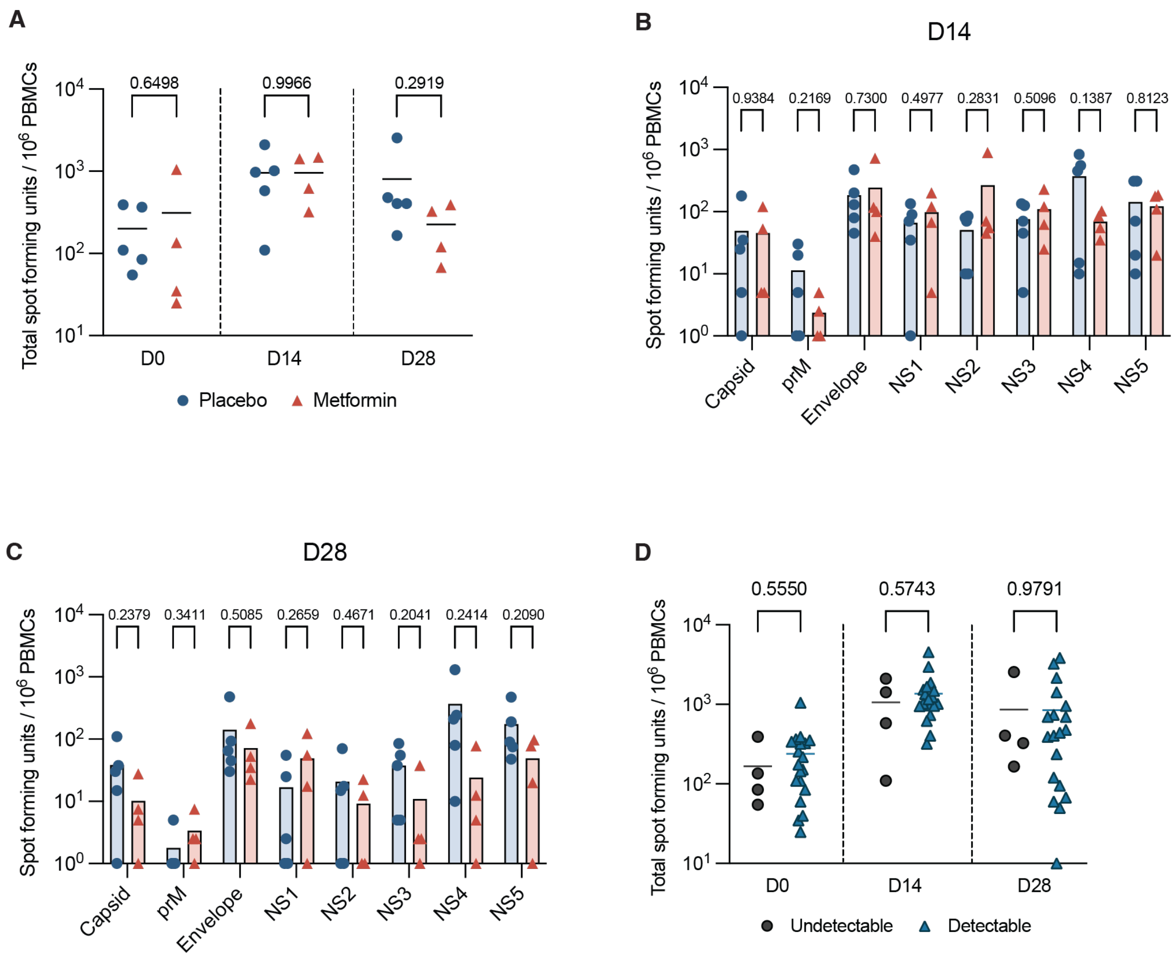

Appendix Figure S4 – IFNγ T cell response against YF17D were not different between placebo and metformin subgroups.

A Total ex-vivo T-cell IFNγ ELISpot responses against the complete YF17D peptidome at D0 (before vaccination), D14, and D28 in the placebo and metformin.

B, C Number of spot forming units (SFU) in placebo and metformin subgroups in response to peptide pools containing peptides from the structural (Capsid, prM, Envelope) and non-structural (NS1, NS2, NS3, NS4, NS5) regions at D14 and D28 post-vaccination.

D Comparison of total ex-vivo T-cell IFNγ ELISpot responses in subjects with undetectable and detectable viremia against the complete YF17D peptidome at D0 (before vaccination), D14, and D28 post-vaccination.

Data information: In (A and D), lines represent the mean. In (B and C), data are presented as bar graphs illustrating all data points. Statistical analyses were performed using unpaired Student's t-test.

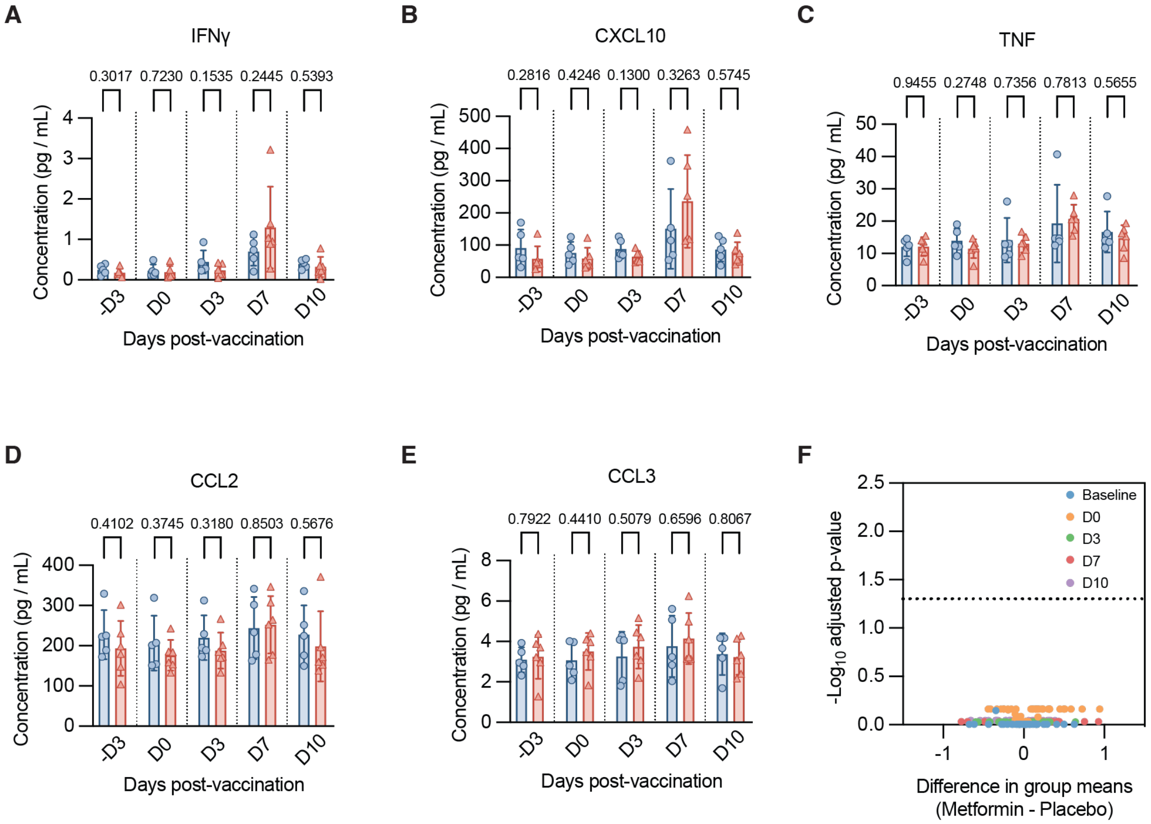

Appendix Figure S5 – Cytokine expression following YF17D vaccination was not significantly different between placebo and metformin subgroups.

A – E Plasma concentrations of IFNγ, CXCL10, TNF, CCL2, and CCL3 at -D3 (baseline), D0 (before vaccination), D3, D7, and D10 post-vaccination in placebo (blue circles) and metformin (red triangles) subgroup.

F Volcano plot illustrating the difference in $\log_2$ mean concentration of 37 cytokines between the metformin and placebo subgroups at the indicated timepoints.

Data information: In (A – E), data are represented as mean ± SD, and statistical analyses were performed using unpaired Student's t-test.

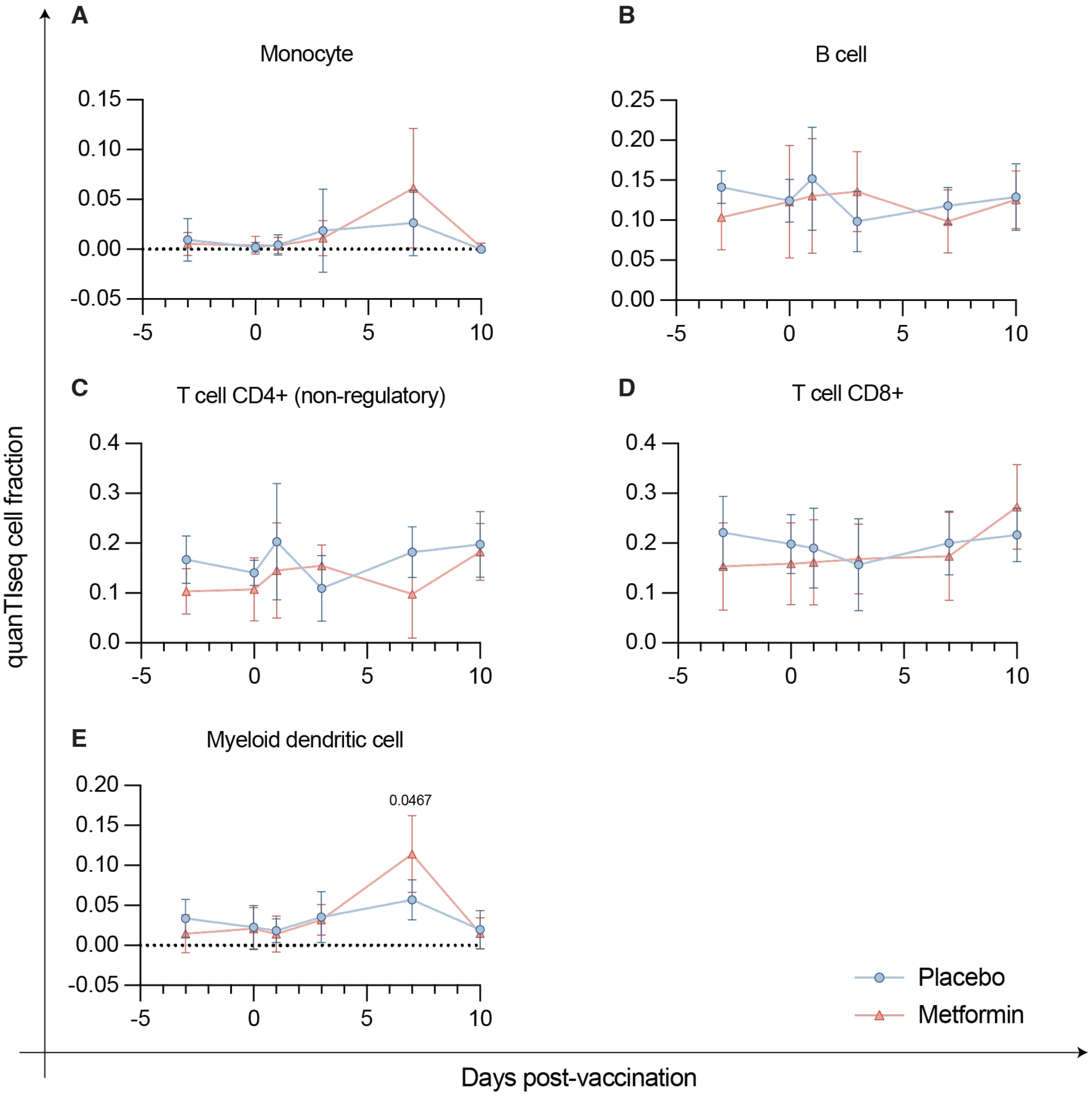

Appendix Figure S6 – Metformin treatment did not alter cell frequency of innate and adaptive immune cells.

A – E Immune deconvolution of bulk RNAseq data illustrating quanTIseq cell fractions of monocytes, B cells, CD4+ T cells, CD8+ T cells, and myeloid dendritic cells at baseline (-D3), before vaccination (D0), D1, D3, D7, and D10 post-vaccination in placebo and metformin subgroups.

Data information: Data are represented as mean ± SD. Statistical analyses were performed using unpaired Student’s t-test.

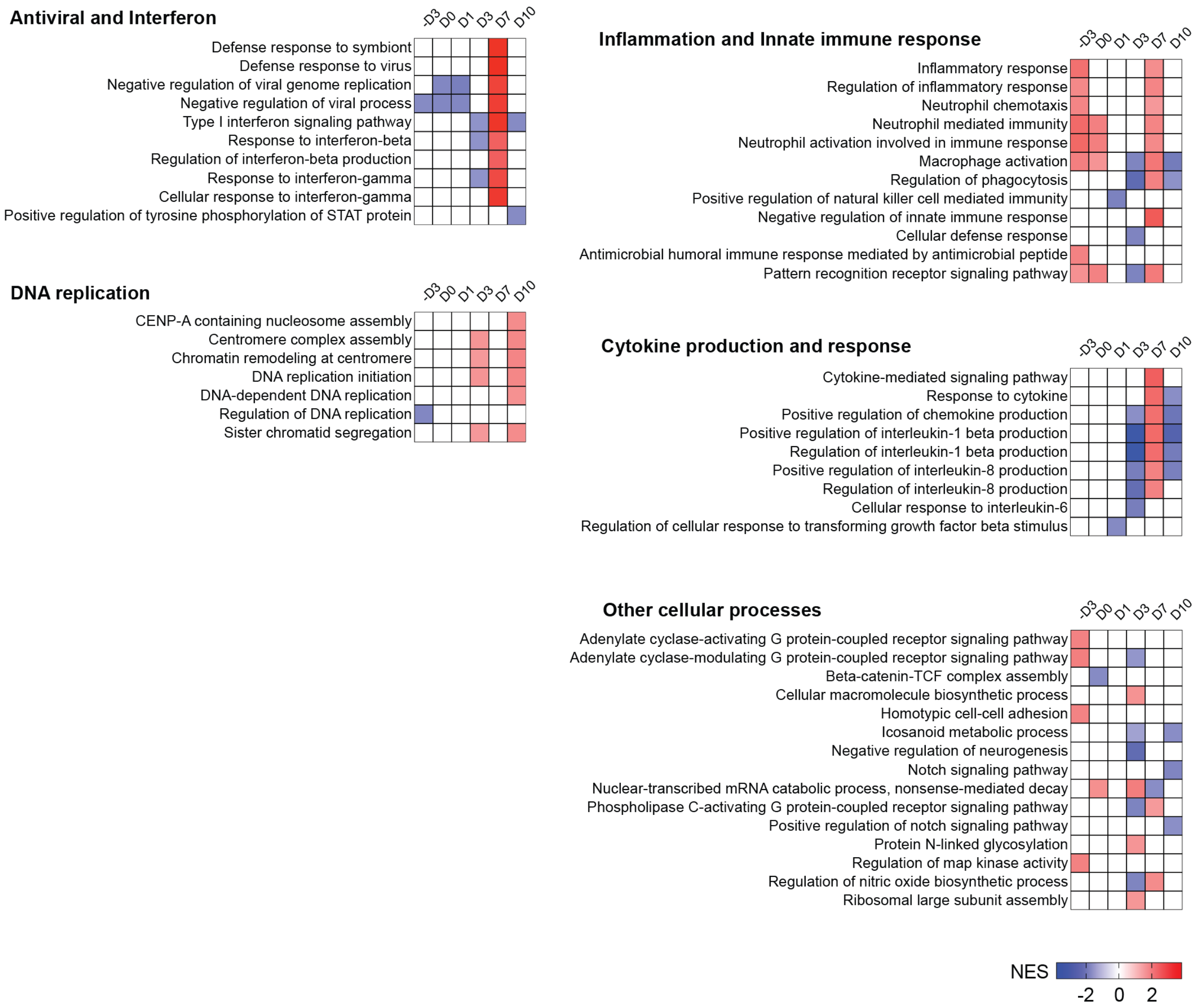

Appendix Figure S7 – GSEA pre-ranking of differentially enriched pathways in metformin vs placebo subgroups.

Gene set enrichment analysis (GSEA) pre-ranking with Gene Ontology Biological Process (GOBP) module at baseline (-D3), before vaccination (D0), D1, D3, D7, and D10 post-vaccination as represented by normalized enrichment scores (NES). Red, blue, and white colors indicate increased, decreased, or no significant difference in enrichment respectively between the metformin and placebo subgroups.

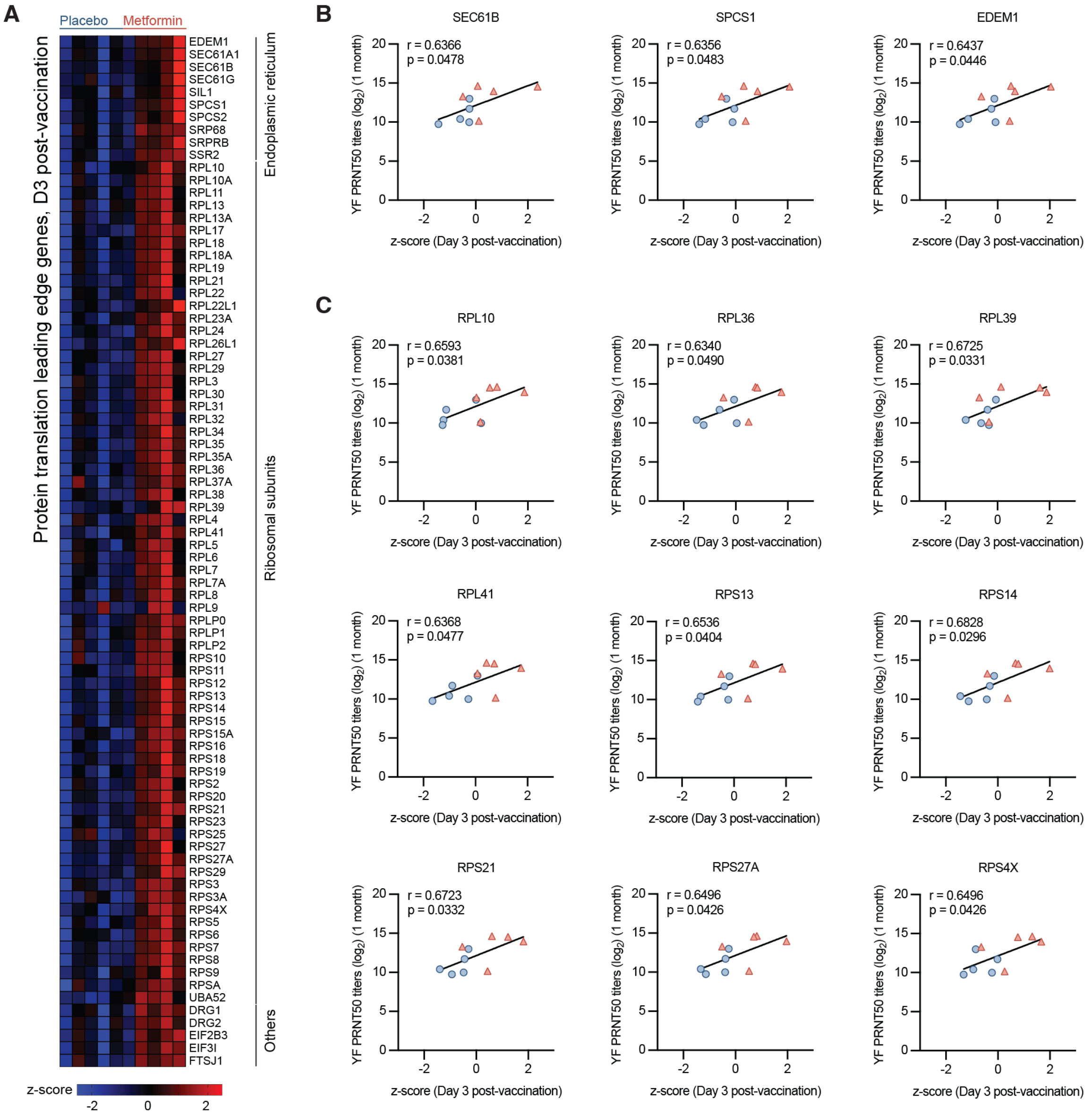

Appendix Figure S8 – Expression levels of endoplasmic reticulum (ER) and ribosomal subunit genes at D3 post-vaccination were correlated with eventual YF17D neutralizing antibody response.

A Z- score normalized expression of leading edge genes (n = 82) in protein translation pathways at D3 post-vaccination.

B, C Pearson correlation of $PRNT_{50}$ titers at 1 month post-vaccination against D3 normalized expression of ER related genes SEC61B, SPSC1, EDEM1, and ribosomal subunit genes RPL10, RPL36, RPL39, RPL41, RPS13, RPS14, RPS21, RPS27A, and RPS4X. Red triangles and blue circles represent the metformin and placebo subgroups respectively.

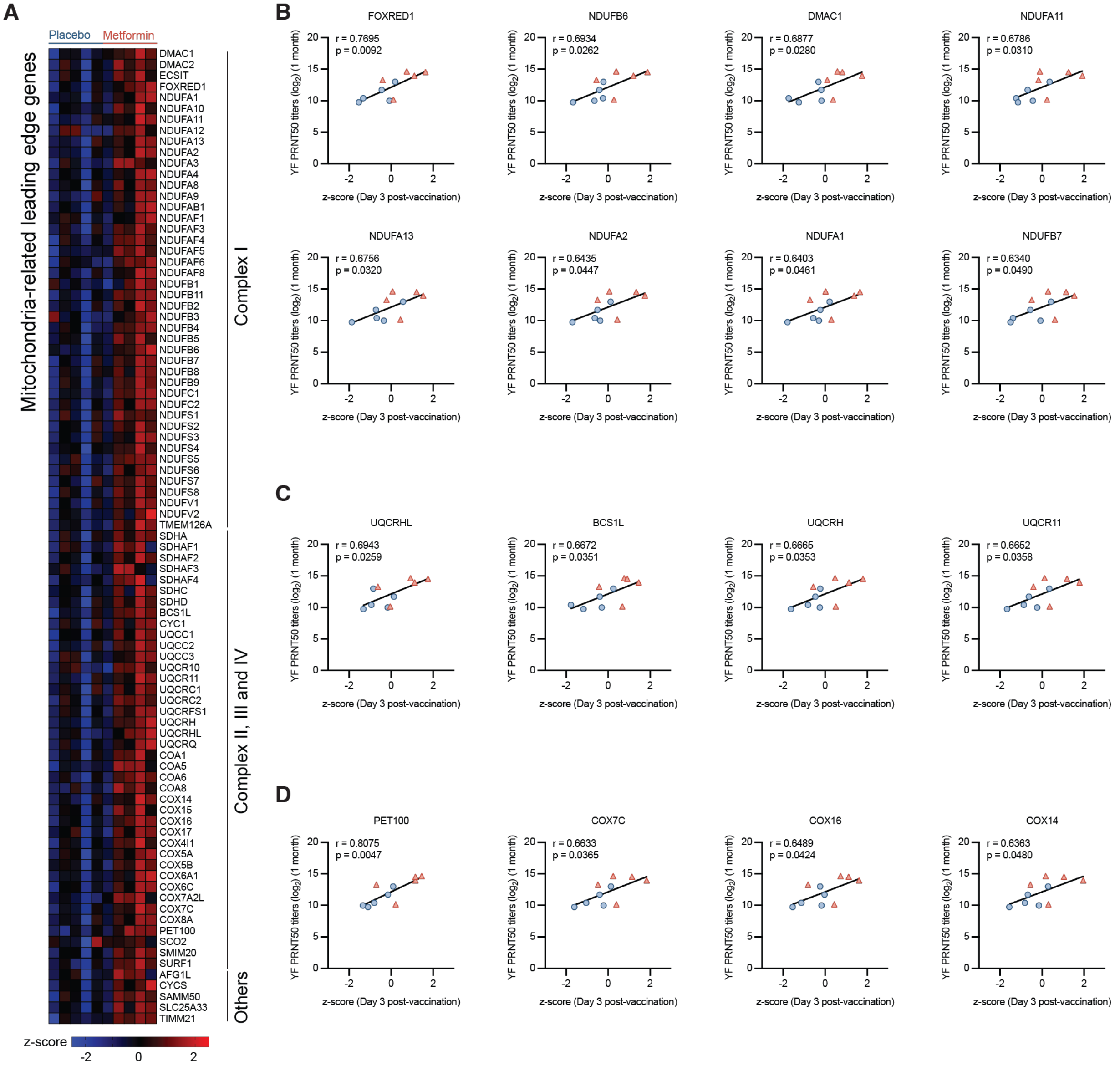

Appendix Figure S9 – Expression levels of mitochondrial respiratory complex genes at D3 post-vaccination were correlated with eventual YF neutralizing antibody response.

A Z-score normalized expression of leading edge genes (*n* = 89) in mitochondria pathways at D3 post-vaccination.

B – D Pearson correlation of YF17D $PRNT_{50}$ titers against D3 normalized expression of complex I, complex III, and complex IV related genes. Red triangles and blue circles represent the metformin and placebo subgroups respectively.

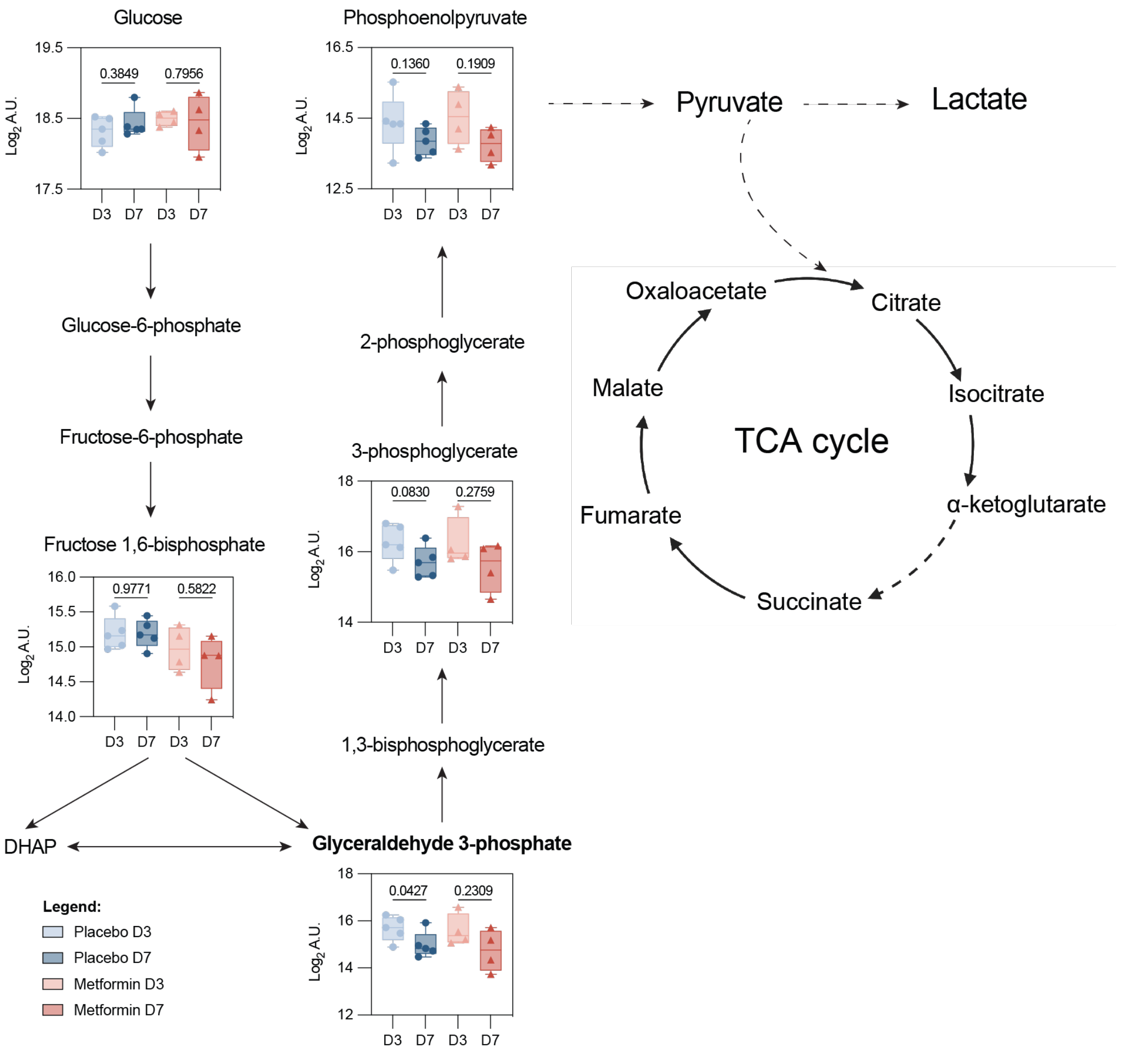

Appendix Figure S10 – Metformin treatment did not alter plasma levels of glucose and glycolytic intermediates upstream of pyruvate.

Plasma level of glycolytic intermediates Glucose, Fructose 1,6-bisphosphate, Glyceraldehyde 3 phosphate, 3-phosphoglycerate, and phosphoenolpyruvate in placebo (n = 5) and metformin (n = 4) subgroups at D3 and D7 post-vaccination. Plasma metabolite levels are represented as $\log_2$ transformed arbitrary units (A.U.).

Data information: Data are represented as box plots showing all data points. Statistical analyses were performed with paired two-tailed t-test.

Appendix Table S1 – Demographics of all recruited subjects

Subject ID	Treatment	Age (years)	Gender	Ethnicity	BMI	DENV IgG Baseline
M01	Metformin	23	Male	Chinese	22.0	Seronegative
M02	Metformin	39	Male	Bruneian	20.5	Seronegative
M04	Placebo	37	Male	Malay	23.6	Seronegative
M07	Placebo	37	Male	Malay	22.3	Seronegative
M09	Metformin	31	Male	Chinese	20.9	Seronegative
M11	Placebo	25	Female	Malay	22.2	Seronegative
M14	Placebo	35	Male	Chinese	22.7	Seronegative
M16	Metformin	30	Male	Chinese	21.2	Seronegative
M18	Placebo	35	Male	German	24.5	Seronegative
M19	Metformin	26	Female	Chinese	21.1	Seronegative
M20	Metformin	34	Female	Chinese	22.3	Seronegative
M21	Metformin	26	Female	Chinese	21.4	Seronegative
M22	Placebo	25	Male	Chinese	23.6	Seronegative
M23	Placebo	35	Male	Chinese	21.4	Seronegative
M24	Placebo	27	Male	Chinese	23.1	Seronegative
M25	Metformin	35	Female	Chinese	21.1	Seropositive
M26	Metformin	37	Female	Chinese	22.4	Seronegative
M29	Metformin	30	Male	Chinese	23.5	Seronegative
M30	Placebo	34	Male	Chinese	24.1	Seronegative
M31	Metformin	35	Male	Chinese	21.1	Seropositive
M32	Placebo	33	Male	Chinese	22.5	Seronegative
M33	Placebo	38	Male	Chinese	21.4	Seropositive
M34	Placebo	32	Male	Chinese	20.0	Seronegative
M35	Metformin	39	Female	Chinese	20.1	Seronegative
M36	Metformin	30	Female	Chinese	21.8	Seropositive
M37	Metformin	24	Female	Indian	23.9	Seronegative

Appendix Table S2 – List of cytokines and chemokines measured

	Cytokine Name
**Chemokines**	CCL11<br>CCL13<br>CCL19<br>CCL2<br>CCL3<br>CCL4<br>CCL7<br>CCL8<br>CXCL10<br>CXCL11<br>CXC12<br>CXCL8<br>CXCL9
**Growth Factors**	CSF1<br>CSF3<br>EGF<br>FLT3LG<br>HGF<br>TGFA<br>VEGFA
**Interleukins and Interferons**	IL10<br>IL15<br>IL17A<br>IL17C<br>IL18<br>IL27<br>IL6<br>IL7<br>OSM<br>IFNG
**Other pro-inflammatory cytokines**	LTA<br>MMP1<br>MMP12<br>OLR1<br>TNF<br>TNFSF10<br>TNFSF12

Appendix Table S3 – List of immunoglobulin genes analyzed

Gene Symbol	Full Name
IGHA1	Immunoglobulin heavy constant alpha 1
IGHA2	Immunoglobulin heavy constant alpha 2
IGHD	Immunoglobulin heavy constant delta
IGHG1	Immunoglobulin heavy constant gamma 1
IGHG2	Immunoglobulin heavy constant gamma 2
IGHG3	Immunoglobulin heavy constant gamma 3
IGHG4	Immunoglobulin heavy constant gamma 4
IGHM	Immunoglobulin heavy constant mu
IGHV3-23	Immunoglobulin heavy variable 3-23
IGHV3-7	Immunoglobulin heavy variable 3-7
IGKC	Immunoglobulin kappa constant
IGKV1-5	Immunoglobulin kappa variable 1-5
IGKV3-11	Immunoglobulin kappa variable 3-11
IGKV3-15	Immunoglobulin kappa variable 3-15
IGKV3-20	Immunoglobulin kappa variable 3-20
IGKV4-1	Immunoglobulin kappa variable 4-1
IGLC2	Immunoglobulin lambda constant 2
IGLC3	Immunoglobulin lambda constant 3
IGLV1-40	Immunoglobulin lambda variable 1-40
IGLV1-44	Immunoglobulin lambda variable 1-44
IGLV1-47	Immunoglobulin lambda variable 1-47
IGLV1-51	Immunoglobulin lambda variable 1-51
IGLV2-8	Immunoglobulin lambda variable 2-8

Appendix Supplemental Information – CONSORT Flow diagram

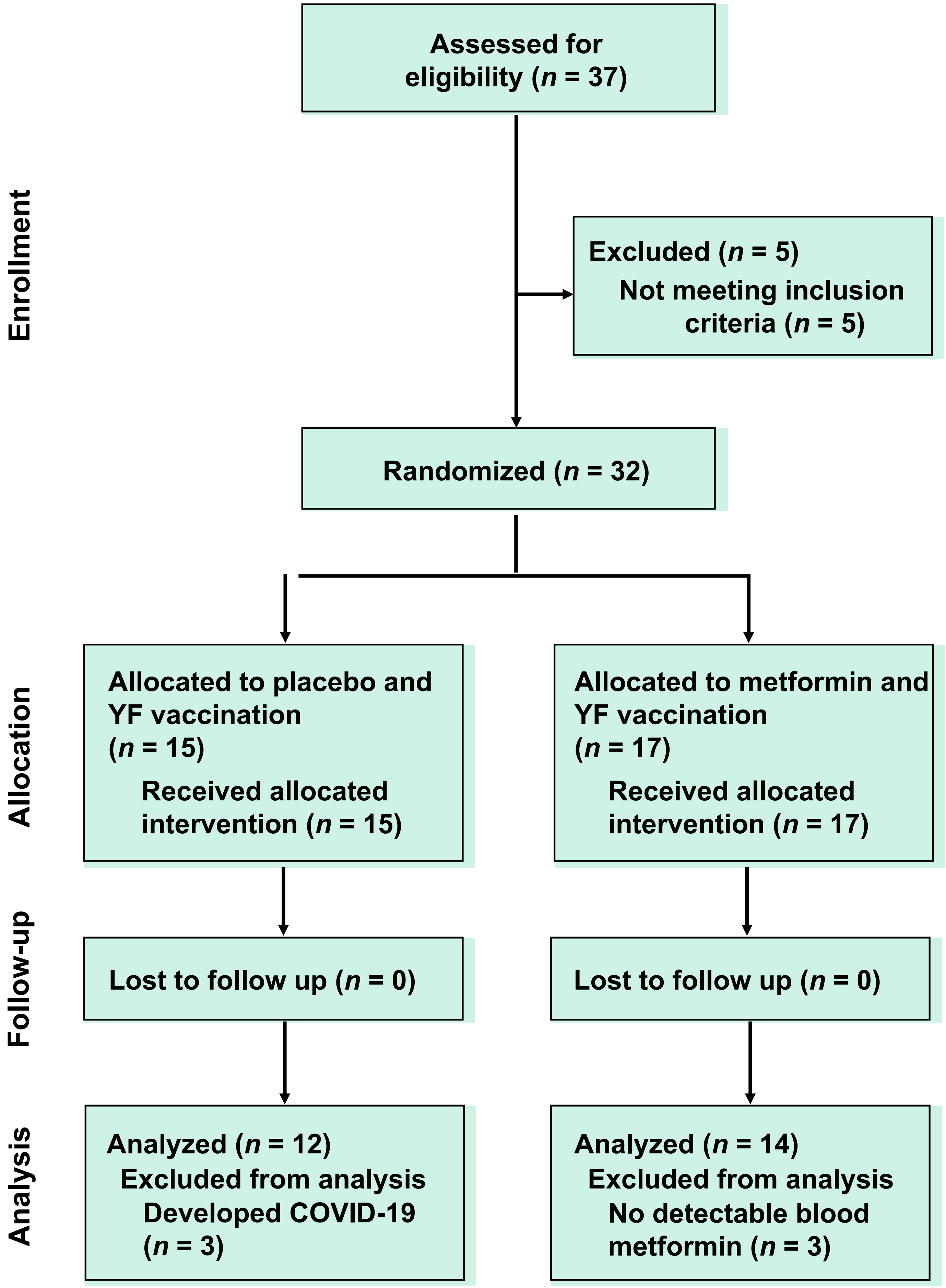

Supplement: Supplementary file 1 — Appendix [file 44321_2024_65_MOESM1_ESM.pdf]
